# Human Immunodeficiency Virus-Associated Nephropathy in Pregnancy

**DOI:** 10.1155/S1064744996000191

**Published:** 1996

**Authors:** Nancy L. Eriksen, Joan M. Mastrobattista

**Affiliations:** Department of Obstetrics, Gynecology, and Reproductive Sciences University of Texas Health Science Center Lyndon B. Johnson Hospital 5656 Kelley Houston TX 77026 USA

## Abstract

*Background:* Human immunodeficiency virus (HIV)-associated nephropathy typically leads to endstage renal disease requiring dialysis within 3–4 months. This report describes the prenatal course of a patient with HIV-associated nephropathy requiring dialysis during pregnancy.

*Case:* A 23-year-old nulliparous, black female presented at 13 weeks gestation with a history of HIV-associated nephropathy and anemia. She had a CD4 count of 350/mm^3^, a total urinary protein of 1.7 g/day, and a serum creatinine of 4.8 mg/dl. The patient was begun on zidovudine, 500 mg daily, and erythropoietin, 4,000 units weekly. At 23 weeks gestation, when she developed hypertension, a total urinary protein of 3.4 g/day, and a serum creatinine of 4.4 mg/dl, she was hospitalized. Her renal function continued to deteriorate, requiring hemodialysis. At 29-4/7 weeks, she developed preterm labor, for which she was placed on indomethacin. Four days later, at 30 weeks gestation, she delivered a viable male infant.

*Conclusion:* HIV-associated nephropathy during pregnancy can be successfully managed with hemodialysis.


Infectious Diseases in Obstetrics and Gynecology 4:89–91 (1996)
© 1996 Wiley-Liss, Inc.

### Human Immunodeficiency Virus-Associated Nephropathy in Pregnancy

**Nancy L. Eriksen and Joan M. Mastrobattista**

*Department of Obstetrics, Gynecology, and Reproductive Sciences, University of Texas Health Science Center, Lyndon B. Johnson Hospital, Houston, TX*

### Abstract

***Background:*** **Human immunodeficiency virus (HIV)-associated nephropathy typically leads to end-stage renal disease requiring dialysis within 3–4 months. This report describes the prenatal course of a patient with HIV-associated nephropathy requiring dialysis during pregnancy.**

***Case:*** **A 23-year-old nulliparous, black female presented at 13 weeks gestation with a history of HIV-associated nephropathy and anemia. She had a CD4 count of 350/mm$^3$, a total urinary protein of 1.7 g/day, and a serum creatinine of 4.8 mg/dl. The patient was begun on zidovudine, 500 mg daily, and erythropoietin, 4,000 units weekly. At 23 weeks gestation, when she developed hypertension, a total urinary protein of 3.4 g/day, and a serum creatinine of 4.4 mg/dl, she was hospitalized. Her renal function continued to deteriorate, requiring hemodialysis. At 29-4/7 weeks, she developed preterm labor, for which she was placed on indomethacin. Four days later, at 30 weeks gestation, she delivered a viable male infant.**

***Conclusion:*** **HIV-associated nephropathy during pregnancy can be successfully managed with hemodialysis.** © 1996 Wiley-Liss, Inc.

Key words

AIDS, kidney, hemodialysis, dialysis, glomerulosclerosis

The incidence of human immunodeficiency virus (HIV) is rising rapidly among women of reproductive age, particularly among black women. Noninfectious complications of HIV, such as nephropathy, can occur. In most cases, this entity rapidly progresses to end-stage renal disease requiring dialysis within 3–4 months.[1] Renal failure during pregnancy, a difficult problem to manage, can result in an increased risk of perinatal loss. This report of HIV-associated nephropathy during pregnancy illustrates the therapeutic issues related to this complication.

### CASE REPORT

A 23-year-old black woman, $G_1P_0$, presented for prenatal care at 13 weeks gestation to the Infectious Disease Clinic at Lyndon Baines Johnson Hospital. The patient, who had been HIV positive for 1 year, denied any history of IV drug abuse.

One year earlier, she had been hospitalized with pyelonephritis, generalized lymphadenopathy, and nephrotic syndrome. An axillary lymph node was suspicious for lymphoma and a biopsy was read as reactive follicular hyperplasia consistent with HIV lymphadenopathy. A computed axial tomogram revealed retroperitoneal lymphadenopathy, and a renal sonogram showed bilateral echogenic kidneys. A kidney biopsy (prior to pregnancy) showed focal segmental glomerulosclerosis. Immunofluorescence studies demonstrated C3 and IgM staining of the sclerotic glomeruli.

Immunofluorescence studies were negative for IgA deposits. The biopsy results were consistent with HIV-associated nephropathy. A Western blot

Address correspondence/reprint requests to Dr. Nancy L. Eriksen, Department of Obstetrics and Gynecology, Lyndon B. Johnson Hospital, 5656 Kelley, Houston, TX 77026.

Received March 22, 1996
Accepted June 14, 1996

confirmed an HIV infection. The patient's CD4 count was 471/mm$^3$ at the time of her initial diagnosis. She had a blood urea nitrogen (BUN) of 44 mg/dl, a serum creatinine of 5.5 mg/dl, and a total urinary protein of 4 g/day. Because she was not expected to survive long with end-stage renal disease, her nephrologist elected not to start antiretroviral therapy with zidovudine. A right-wrist arterial venous fistula was created 4 months later in anticipation of dialysis. The patient was subsequently lost to follow-up for approximately 6 months.

At her initial prenatal visit, her blood pressure was 117/60. She had a hemoglobin of 9.8 mg/dl and hematocrit of 27.7%. Her CD4 count was 380 mm$^3$ and serum creatinine was 4.8 mg/dl, with a BUN of 52 mg/dl. The total urinary protein was 1.7 g/day and serum albumin was 2.7 mg/dl. She was begun on multivitamins; ferrous sulfate; recombinant erythropoietin, 4,000 units weekly; and zidovudine, 500 mg daily. The nephrology service was consulted, and her renal function was serially assessed by evaluations of serum creatinine and BUN and 24-h urine collections for total protein and creatinine clearance. Ultrasounds at 10 and 20 weeks gestation were consistent with her menstrual dates, and no fetal anomalies were noted.

Her prenatal course was uncomplicated until 23 weeks gestation when her blood pressure increased to 143/92. She had a total protein of 3.4 g/day and creatinine clearance of 15 ml/min. Her serum creatinine was 4.4 mg/dl. Her anemia was slightly worse, with a hemoglobin of 8.3 mg/dl and hematocrit of 22.8%. The clinical picture was suspicious for either worsening renal disease or mild superimposed preeclampsia. After she was hospitalized, her blood pressure improved slightly at bed rest. However, her renal function continued to deteriorate over the ensuing 8 days. Worsening azotemia and increasing serum creatinine of 5.8 mg/dl were noted and hemodialysis was begun. She tolerated hemodialysis well, with minimal maternal hypotension and rare fetal heart-rate decelerations. Dialysis was performed 3 times per week to maintain a predialysis BUN of <50 mg/dl. The patient was discharged from the hospital at 26 weeks gestation and closely followed as an outpatient.

At 29-6/7 weeks, the patient complained of vaginal bleeding. An examination revealed the cervix to be 3 cm dilated and 1 cm long, with regular uterine contractions every 5 min. Successful tocolysis was achieved with indomethacin, and betamethasone was administered to enhance the fetal lung maturity. Four days later, labor ensued, and she received intrapartum IV zidovudine. A viable male infant was spontaneously delivered at 30 weeks gestation. The infant weighed 1,360 g, with Apgars of 8 at 1 and 5 min.

Her postpartum course was unremarkable. Her blood pressure returned to baseline at her postpartum visit. For 24 months, she has been maintained on hemodialysis, 3 times per week, and zidovudine, 500 mg day, with no opportunistic infections to date. Her infant is HIV seronegative at 2 years of age.

### DISCUSSION

HIV-associated nephropathy was first reported in 1983. A patient with this complication typically presents with proteinuria (>3.5 g/day) and hypoalbuminemia. More than 95% of all cases of HIV-associated nephropathy are found in blacks, with a male-to-female ratio of 10:1.[1] Approximately 50% of patients with HIV-associated nephropathy acquired HIV infection through IV-drug abuse, while the remaining cases acquired it through sexual contact.[1]

The predominant pathologic lesion in HIV-associated nephropathy is focal segmental glomerulosclerosis, which accounts for over 90% of the histologic changes.[2] The characteristic features of HIV-associated focal segmental glomerulosclerosis are qualitatively similar to other forms of focal segmental glomerulosclerosis, including cases attributable to drug abuse and idiopathic cases.

While the time required for the development of nephropathy in adults is not known and the initial time of the acquisition of HIV infection is difficult to determine, the onset of proteinuria in children ranges from 2.5 to 4.9 years.[1] HIV-associated nephropathy results in a rapid loss of renal function that leads to end-stage renal disease, typically within 4 months, although wide variations in the time course are seen. Once dialysis becomes necessary, the survival is generally <6 months.[3] Recent data indicate that the clinical stage of HIV infection may be the most important determinant of survival. Ortiz et al.[4] found the mean duration of dialysis to range from 74 days in acquired immunodeficiency syndrome (AIDS) patients to 255 days in asymptomatic patients. These data suggest that maintenance dialysis should be performed only in cases of asymptomatic HIV infection. In 1993, the diagnosis of AIDS was assigned to anyone with a CD4 count of <200 min[3]; therefore, asymptomatic AIDS patients should also be considered for maintenance hemodialysis.

End-stage renal disease during pregnancy is associated with a poor perinatal outcome, particularly for a patient with primary glomerulonephritis. Focal segmental glomerulosclerosis has a higher rate of perinatal loss (23%) and preterm labor (32%) than other types of glomerulonephritis, presumably because of the greater prevalence of renal insufficiency.[5] A review of 24 pregnant patients undergoing dialysis after 1990 showed that 52% of the pregnancies resulted in surviving infants.[6] Additionally, 79% of these women delivered after 28 weeks gestation, although only 1 infant was delivered at term.

In cases of preterm labor, indomethacin has been used. It appears to be well tolerated for short courses of therapy. Recombinant erythropoietin in pregnancy is also well tolerated.[6,7] Although it has not received U.S. Food and Drug Administration approval for use during pregnancy, theoretically, it should not cross the placenta since it has a molecular weight of 30,400 daltons.[7] In a small series of pregnant women on dialysis, the response to erythropoietin was diminished.[7] Iron-deficiency anemia is the most common reason for resistance to erythropoietin, indicating the advantage of placing pregnant women with end-stage renal disease on iron therapy early. Our patient, who responded well to erythropoietin, did not require any increase in dosage during the pregnancy.

Our patient was offered the AIDS Clinical Trial Group 076 regimen because of the beneficial decrease in maternal-fetal transmission of HIV infection. A full dose of zidovudine (500 mg/day) was given, as very little is excreted renally. Of note, uncontrolled observations suggest that zidovudine can retard or attenuate progression to end-stage renal disease and that discontinuation of zidovudine can lead to a more fulminant clinical course.[8]

A pregnancy complicated by HIV-associated nephropathy requiring dialysis can result in a viable infant despite the increased risk of preterm delivery, as our case demonstrates. Recombinant erythropoietin and zidovudine, both of which are well tolerated, should be considered in the management of such a patient.

### REFERENCES

1. Rao TK: A decade of human immunodeficiency virus-associated nephropathy. Transplant Proc 25:2439–2440, 1993.
2. Bourgoignie JJ: Renal complications of human immunodeficiency virus type 1. Kidney Int 37:1571–1584, 1990.
3. Carbone L, D'Agati V, Cheng JT, Appel GB: Course and prognosis of human immunodeficiency virus-associated nephropathy. Am J Med 87:389–395, 1989.
4. Ortiz C, Meneses R, Jaffe D, Fernandez JA, Perez G, Bourgoigne JJ: Outcome of patients with human immunodeficiency virus on maintenance hemodialysis. Kidney Int 34:253–284, 1988.
5. Imbasciati E, Ponticelli C: Pregnancy and renal disease: Predictors for fetal and maternal outcome. Am J Nephrol 353–362, 1991.
6. Hou SH: Frequency and outcome of pregnancy in women on dialysis. Am J Kidney Dis 23:60–63, 1994.
7. Hou S, Orlowski J, Pahl M, Ambrose S, Hussey M, Wong D: Pregnancy in women with end-stage renal disease: Treatment of anemia and preterm labor. Am J Kidney Dis 21:16–22, 1993.
8. Michel C, Dosquet P, Ronco P, Mougenot B, Viron B, Mignon F: Nephropathy associated with infection by human immunodeficiency virus: A report on 11 cases including 6 treated with zidovudine. Nephron 62:434–440, 1992.
